# Hepatic actinomycosis: a very rare form of actinomycosis

**DOI:** 10.1590/0037-8682-0289-2020

**Published:** 2021-03-08

**Authors:** Ayse Albayrak, Zulal Ozkurt, Kemalettin Ozden

**Affiliations:** 1 Ataturk University, Faculty of Medicine, Department of Infectious Diseases and Clinical Microbiology, Erzurum, Turkey.

**FIGURE 1:**
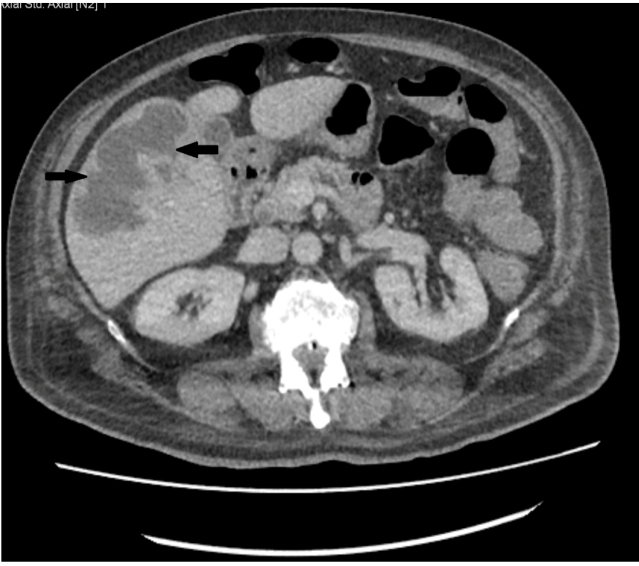
Abdominal computed tomography: an irregular limited lesion area with a contoured lobule measuring 110 × 60 mm in the right lobe of the liver (arrows).

A 72-year-old man presented to the emergency department with a history of fever, general malaise, anorexia, and weight loss of 5-6 kilos over the last 3-4 months. He was diagnosed with diabetes mellitus before 7 years. He had no history of abdominal surgery or gastrointestinal intervention. On physical examination, his body temperature was 39.5°C and his abdomen was soft and non-tender. Abdominal ultrasonography revealed a heterogeneous hypoechoic lesion area measuring 90 × 50 mm in the right lobe of the liver. Abdominal computed tomography showed an irregular limited lesion (Figure 1). The patient had fever (39°C-40°C) 2-3 times a day with severe chills, after which cultures were performed and empiric ceftriaxone 2 g/day (IV) was initiated.

**FIGURE 2:**
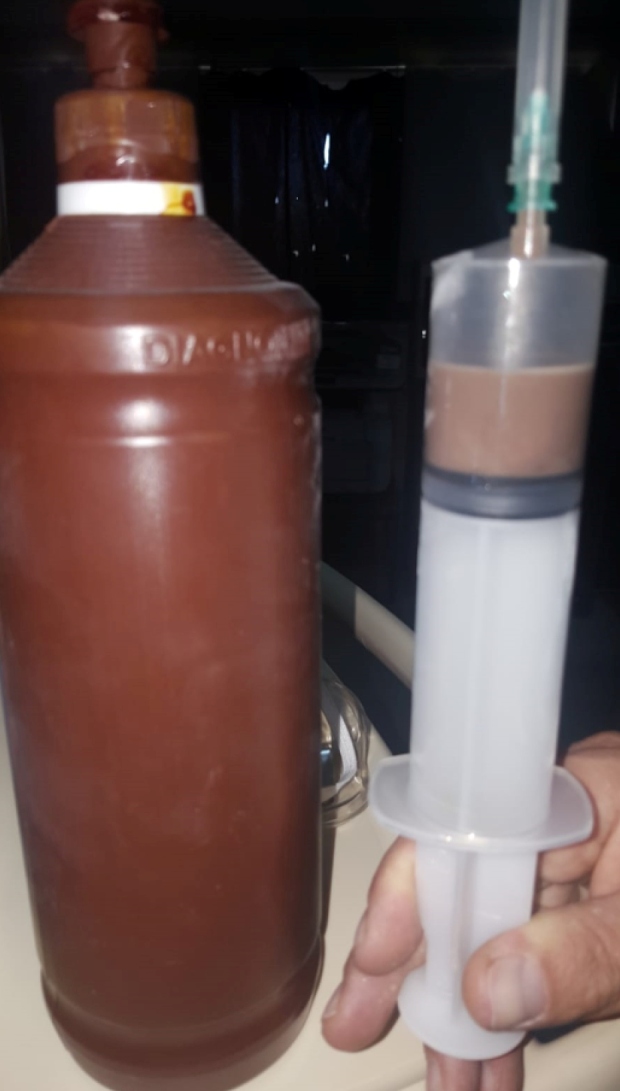
The abscess material.

**FIGURE 3:**
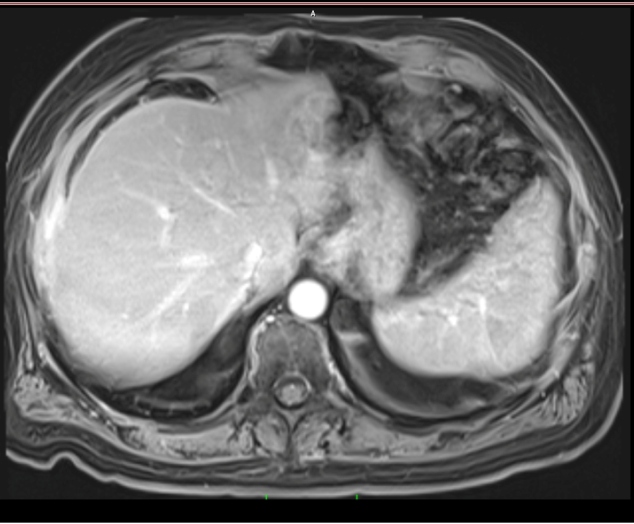
Abdominal magnetic resonance imaging revealed no abnormal findings in the liver.

The hepatic abscess was evacuated (Figure 2). Cytopathological examination revealed neutrophils, histiocytes, and *Actinomyces* colonies. The patient was treated with intravenous penicillin G for 45 days and was discharged on treatment with oral ampicillin. The follow-up abdominal magnetic resonance imaging after 2 months revealed no abnormalities (Figure 3). The treatment was completed in 6 months.

Actinomyces are anaerobic or microaerophilic gram-positive filamentous bacteria that normally colonize the mouth, colon, and vagina^1^ ^,^ ^2^. Hepatic involvement is present in 5% cases of actinomycosis. While hepatic involvement may be seen in disseminated actinomycosis, isolated involvement is most likely due to a hematogenous spread from an unknown focus. 

In the differential diagnosis of liver abscesses, isolated liver actinomycosis should also be considered in patients without a history of abdominal surgery.
